# Supplementary material for: A novel role for Ets4 in axis specification and cell migration in the spider Parasteatoda tepidariorum
Source: eLife. 2017 Aug 29;6:e27590. doi: 10.7554/eLife.27590 (PMC5574703; doi:10.7554/eLife.27590)
Supplement: Supplementary file 1. — DOI: http://dx.doi.org/10.7554/eLife.27590.022 [file elife-27590-supp1.docx]

EGFP-Pt-Ets4-PolyA fusion construct (synthesized at Eurofins Genomics)

>EGFP-Pt-Ets4-PolyA (in pUC57)

ACTAGTTAATACGACTCACTATAGGGCATTTAGGTGACACTATAGGAAGCTTCCTTGTTCTTTTTGCAGAAGCTCAGAATAAACGCTCAACTTTGGCAGATACCGGCCGGCCCTCGAGGGATCCTACGTAATGGTGAGCAAGGGCGAGGAGCTGTTCACCGGGGTGGTGCCCATCCTGGTCGAGCTGGACGGCGACGTAAACGGCCACAAGTTCAGCGTGTCCGGCGAGGGCGAGGGCGATGCCACCTACGGCAAGCTGACCCTGAAGTTCATCTGCACCACCGGCAAGCTGCCCGTGCCCTGGCCCACCCTCGTGACCACCCTGACCTACGGCGTGCAGTGCTTCAGCCGCTACCCCGACCACATGAAGCAGCACGACTTCTTCAAGTCCGCCATGCCCGAAGGCTACGTCCAGGAGCGCACCATCTTCTTCAAGGACGACGGCAACTACAAGACCCGCGCCGAGGTGAAGTTCGAGGGCGACACCCTGGTGAACCGCATCGAGCTGAAGGGCATCGACTTCAAGGAGGACGGCAACATCCTGGGGCACAAGCTGGAGTACAACTACAACAGCCACAACGTCTATATCATGGCCGACAAGCAGAAGAACGGCATCAAGGTGAACTTCAAGATCCGCCACAACATCGAGGACGGCAGCGTGCAGCTCGCCGACCACTACCAGCAGAACACCCCCATCGGCGACGGCCCCGTGCTGCTGCCCGACAACCACTACCTGAGCACCCAGTCCGCCCTGAGCAAAGACCCCAACGAGAAGCGCGATCACATGGTCCTGCTGGAGTTCGTGACCGCCGCCGGGATCACTCTCGGCATGGACGAGCTGTACAAGGGAGGCAGCGGCGGCGGAAGCGGAGGCTCCGGACTCAGATCTCGAGCTCAAGCTTCGATGCAGACGTGTGCTCCCATTCGAGTTGAAACTAGGCCCCAGTTAGTTCCCAATCCAGGAAGTCCGCTGTCCGAATTAGAAGGGTGGACAATGGCCGATTTTGAGAGCTGGCTCTGTGATCCCTCTACTCTGCTCAGAAGTAACAGTAATTTAATGTACACAGCACCTTCTATTATGGAACCACAAAAGATGGCCGCTTATGGCAATGTGCATGTAAGCATTCCATCTCCTTCCGGAGTGCCTTCAGCGGCTGAAAGCCCAACTCTATACGACAGTCCACCAGGTAAAGGCTATGTAGGTCCACCTCCCTATGTCCAGATTAAACAGGAGGCTGAAGAAATTGATTACCAGTGTCCTGGATTACATATGGGACCCATGAGTCCAGACATGAATGTCAAGCACGAAGATGAAGACGATAAAATGGAACAATTGAGAAACATGGCAATGGAACAAGCAGCAAAGGATATTCGTGTTGCTTGCGGCATACTTGGTATCTGTCCTGATCCAACTTTGTGGACAATAGATGAAGCAAAGTCTTGGTTATTATGGATTTTAAACCAATATGGAATGAACACTGAGGTTTTGCAATATTTCAATATGGATGGCTTGGGATTATGCGCTCTATCAGAAGATTACTTCAGACAAAAAATTCCAAATGGTGGAGATATACTATACGCACAACTAGACATCTGGAAAACTGCATCTAGCCTTACATGCCAACCTCCAAATCCTCCACGCCAACAACAATCCCTGCTATCATTTCGACCAGAGGACAGCATGCTGGACATGAGCATCCTTGATCAATGGTCACCTTATCACCAGCAACAACTTCGCATGTCACCCCCGGCTGGTTCCAGGGGTGGCACCATGGTTGCAATTCCTGACAGTAGCAGTTCTGTTGCATCACCAGAAAGTAGCCATCATGATTTCAGCAGTGAAGGAATACAGAGTGATGATGATATAAGCGATGAATCCTGTGACGTTGAGCGTTGCGGGGGTGCCAGAACAGGAGGTACCAGCGGAGGCCGCCCCGGTTCCCACTCACACATCCATCTCTGGCAATTCCTGAAAGAGCTCTTGTGCCAATCCCACTTATACGGCAGTTGCATACGATGGTTGGACAGACCAAAGGGCATCTTCAAGATCGAAGACTCAGTCCGAGTTGCTAGGCTCTGGGGAAAGAGGAAGAACAGGCCAGCAATGAACTACGACAAACTCAGCCGTTCCATTCGCCAATATTACAAGAAGGGGATCATGAAAAAGACTGAAAGGTCTCAGAGACTGGTGTACCAGTTCTGTCACCCTTACGGTCTCTAGAGATCTTACGTAAGTACTGGGTCGACCTTAAGGGCGCGCCCGGCCGCGACTCTAGAGGATCTGGTTACCACTAAACCAGCCTCAAGAACACCCGAATGGAGTCTCTAAGCTACATAATACCAACTTACACTTTACAAAATGTTGTCCCCCAAAATGTAGCCATTCGTATCTGCTCCTAATAAAAAGAAAGTTTCTTCACATTCTAAAAAAAAAAAAAAAAAAAAAAAAAGCGGCCGCCCATGGCTGCAGGAGCTCGAATTCACTAGT

T7 promoter

Sp6 promoter

5’ and the 3’ UTR of the *Xenopus* beta-globin gene

EGFP

GS-linker

Pt-Ets4

PolyA
